# What are the essential cognitive requirements for prospection (thinking about the future)?

**DOI:** 10.3389/fpsyg.2014.00626

**Published:** 2014-06-30

**Authors:** Magda Osman

**Affiliations:** Biological and Experimental Psychology Group, Queen Mary University of LondonLondon, UK

**Keywords:** prospection, episodic memory, causality, future thinking, planning, goal-directed behavior, comparitive cognition

## Abstract

Placing the future center stage as a way of understanding cognition is gaining attention in psychology. The general modern label for this is “prospection” which refers to the process of representing and thinking about possible future states of the world. Several theorists have claimed that episodic and prospective memory, as well as hypothetical thinking (mental simulation) and conditional reasoning are necessary cognitive faculties that enable prospection. Given the limitations in current empirical efforts connecting these faculties to prospection, the aim of this mini review is to argue that the findings show that they are sufficient, but not necessary for prospection. As a result, the short concluding section gives an outline of an alternative conceptualization of prospection. The proposal is that the critical characteristics of prospection are the discovery of, and maintenance of goals via causal learning.

## Introduction

Prospection refers to the process of representing and planning for possible future states of the world (Buckner and Carroll, 2007; Gilbert and Wilson, 2007; Suddendorf and Corballis, 2007; Seligman et al., 2013). Several recent reviews in human psychology (e.g., Schacter et al., 2008; Szpunar, 2010; Seligman et al., 2013), along with an ensuing debate in comparative psychology have helped draw attention to the role of prospection in cognition (animal and human) (e.g., Raby and Clayton, 2009; Roberts and Feeney, 2009; Suddendorf et al., 2009; de Waal and Ferrari, 2010). What this mini review does differently to extant reviews is to: (1) describe current theoretical thinking on the necessary cognitive requirements of prospection, (2) discuss the empirical problems concerning the necessary cognitive requirements of prospection, and (3) propose an alternative conceptualization of prospection.

### Point 1: cognitive requirements of prospection

Many theorists (Buckner and Carroll, 2007; Gilbert and Wilson, 2007; McDaniel and Einstein, 2007; Schacter et al., 2007; Suddendorf and Corballis, 2007) claim that episodic memory and prospective memory are essential for prospective cognition. *Episodic memory* is most commonly defined as autobiographical memory of experiences that encapsulate thoughts, emotions, motivations, perceptions and beliefs at the time of a personally experienced event. These types of memories provide the informational content that enables simulation of future experiences and outcomes. By extension, *prospective memory is* the tagging of intentions onto episodic representations; the intentions to act may connect to a specific future context (event-based prospective memory) or to a specific future time interval (time-based prospective memory) (McDaniel and Einstein, 2007). Gollwitzer (1999) refers to the time and context as implementation intentions whereas goal intentions contain details of the desired outcome of the intended action. Critically, all prospective memories are retrieved in the absence of external reminders. This means that in order to execute the intended action, people must keep active in memory conditional representations (e.g., if context/time X occurs, I must perform action Y) (Gollwitzer, 1999). In order to form representations of the future an individual needs to deconstruct and recombine currently held representations in order to form expectations of possible outcomes. For this reason Gilbert and Wilson (2007) propose that *hypothetical thinking* is essential for prospective thinking, this is because it is a process by which symbols can be manipulated in such a way as to enable prospective thinking. Hypothetical thinking is a means to inferring and imagining the emotional, motoric, and sensory details of events that are fantastical (e.g., riding a unicorn) or unfamiliar (e.g., being President of the United States). In parallel to this, Seligman et al. (2013) emphasize the role of reasoning in prospection by proposing that prospective thoughts are structured as *conditionals* (e.g., if X, then Y). As a result, in order to think prospectively, the essential requirements are to construct and evaluate conditional propositions. However, others propose that conditional reasoning is a pre-requisite for hypothetical thinking, rather than simply accompanying it (Rescher, 1961). It is also worth noting that Seligman et al.'s (2013) proposals dovetail Gollwitzer's (1999) claims concerning prospective memory because implementation intentions are structured in the form of conditionals.

### Point 2: problems and empirical challenges

Given the strong theoretical claims concerning the necessary components of prospection, the following section critically examines the evidence for these claims.

#### Episodic memory and prospective memory

Evidence in support of the essential role of episodic memory in prospective thinking comes in two forms. One is in the form of neuro-imaging studies. If regions in the brain are associated with episodic memory then they should become active when prospective thinking is engaged, which is indeed the case (Spreng and Grady, 2010; Gerlach et al., 2011; Cooper et al., 2013). For instance, Addis et al.'s (2007) study involved scanning participants while they imagined themselves experiencing an event based on cued words (e.g., star, dress) and a specified time horizon (e.g., past week, next week, past year, next year), and asking them to describe the events after being scanned. Addis et al. (2007) found that the region associated with episodic memory (i.e., medial left pre-frontal parietal) was also active during imagining of past and future events.

The other form of support is based on clinical studies. The idea being patients with impaired episodic memory should also show impaired prospective thinking, which has also found support (Klein et al., 2002; Race et al., 2011; Irish et al., 2012). When Klein et al. (2002) compared patient D. B. (aged 78) with severe anterograde amnesia with two healthy age matched controls, D. B. showed impaired performance on tests of recollection of past experiences, as expected, but inaccurate responses to test of prospective memory (e.g., How will you spend next Christmas?).

However, when compared to healthy age-matched controls, the evidence from clinical studies of patients with Autism, Schizophrenia, Depression, and Impulse-control problems also reveals impairments in prospective thinking (Wallace, 1956; O'Connor et al., 2000; MacLeod et al., 2005; Bechara et al., 2006; D'Argembeau et al., 2008; Lind and Bowler, 2010; Griffiths et al., 2012). These findings are problematic for several reasons. For a start, not all of these clinical populations have impairments to episodic memory, and they don't all show the same deficit in prospective thinking; this may be due to the fact that the measures of prospection are not consistently the same across studies. If there are any consistent patterns across studies they indicate that patients have difficulty in generating examples of prospective positive events as compared to generating prospective negative events.

The focus here has been on episodic memory because most theorists have made stronger claims concerning its essential role in prospection. Nevertheless, there is an amassing literature on prospective memory which has demonstrated its close connections with the execution of future actions of intentions to act out simple behaviors (see McDaniel and Einstein, 2007). As with episodic memory, the clinical studies on impairments to prospective memory paint a complex and mixed picture. A recent review of clinical studies of patients with Schizophrenia (Ordemann et al., 2014) showed that impairments in prospective thinking are better explained by lifestyle factors than neuropsychological factors. This strongly implies that there is a complex interconnected set of factors that underpin prospective cognition that are no yet fully understood.

#### Hypothetical thinking

As discussed earlier, connections have been drawn between episodic memory and hypothetical thinking (Buckner and Carroll, 2007; Baird et al., 2011). The proposed argument is that evidence for the association between episodic memory and prospective thinking in turn implies an association between hypothetical thinking and prospection. However, this is rather an indirect form of evidence for a link between hypothetical thinking and prospection. In addition, hypothetical thinking is also thought to include mind-wandering, imagining, and mental simulation—which again provides indirect evidence for an association between hypothetical thinking and prospection (Van Boven et al., 2009; Christian et al., 2013). Attempts to demonstrate associations between imagining and prospection are in their infancy, and as yet the evidence is unconvincing. For instance, Christian et al. (2013) presented participants with a mental simulation task. The critical manipulation involved either imagining activities happening next week, or 10 years from now. Christian et al. (2013) found that, for the proximal time scale, the degree of detail of the drawing of the activities varied according to how involved the activity was. While the aim was to show that the content of imagination is affected by what we are expected to do in the future, the findings failed to show that there was any difference in the details when thinking about different activities into the distant future; suggesting that imagination may be weak when it comes to thinking prospectively far into the future.

#### Conditionals

The general claim by Seligman et al. (2013) is that expectations and predictions of future events are structured in the form of conditionals, and by the same token, thinking of future events must involve conditional reasoning. There is however limited work to support this. De Brigard and Giovanello (2012) compared descriptions of positive or negative events that had either happened (episodic memories), could happen (future events in the form of conditionals) and could have happened (in the form of counterfactuals). They found that there were more sensory and spatial details in the episodic memories than the other two types of descriptions. However, there was no difference between the details for future and counterfactual events, which suggests that conditionals concerning future events don't *per se* take on a different status or are processed differently as compared to counterfactuals.

With the exception of some neuroimaging evidence and some work in prospective memory, all other claims concerning the apparent necessary connection between the core cognitive requisites (i.e., episodic memory, prospective memory, hypothetical thinking, conditional representations), and prospection exceed the evidence. Moreover, no evidence thus far suggests that these cognitive processes have a core functional basis in prospective thinking. In addition, the methods used to examine prospective thinking appear to be limited to imagining future events, with the exception of prospective memory which often involves simple reaction time tasks. Thus, far, none of the studies examine the influence of goals (goal development/goal discovery) on prospective thinking. They also don't examine planning behaviors for future events, and they do not test causal knowledge which would be involved in establishing how future events would be brought about (with the exception of theoretical work by Gollwitzer, 1999). Therefore, it appears that the claims made thus far can only demonstrate that episodic memory, prospective memory, hypothetical thinking, and conditional representations are sufficient, but not necessary for prospection.

### Point 3: an alternative conceptualization of prospection

The main thrust of the alternative conceptualization that I will propose here is designed to tackle a critical gap highlighted in the previous section concerning research on prospection, and that is examining the role of planning and causal learning in prospection; both of which are under-researched, but also critical to prospective cognition. Why? The key claim I make (see also Osman, 2014) is that goals are by definition prospects. By exploring our environment we learn to discover ways of maintaining what we need (food, water, shelter), but also extended our reach (developing new skills and knowledge), both of which require representations of the future in the form of goals. Our ability to construct representations of the future and adapt to future outcomes is built on basic contingency learning mechanisms that generate expectations and plans of actions. Expectations and plans of action are guided by the discovery of, and maintenance of goals (e.g., Kempton, 1986; Osman, 2010, 2014; Pezzulo and Rigoli, 2011). We are able to reach our goals by learning to associate planned actions with intended outcomes (Strathman et al., 1994; Karniol and Ross, 1996). Thus, the core argument proposed here is that, if goals are prospects, and contingency learning connects actions with intended outcomes guided by goals (a view endorsed by Gollwitzer, 1999 and Seligman et al., 2013), then planning and causal learning are essential properties of prospection, because they enable us to represent how to act in the future. The strong claim being made here is that accurate representations of the future will depend on the accuracy of causal representations that support the planned actions needed to achieve a future goal. One prediction to come from this framework would be that the more people unpack their causal beliefs and the consequences of their actions in achieving future goals, the higher their judgments of likelihood will be of achieving the goals in the future, which is also in line with Tversky and Kohler's (1994) Support Theory.

Finally, the debate in the comparative literature surrounding prospection is based on whether or not the four cognitive components reviewed here are either unique to humans or not; if they are, then only humans possess prospective cognition. This review has argued that the evidence suggest that the four core faculties are not necessary for prospection. Instead alternative faculties such as contingency learning, which non-humans animals possess, may underpin prospective cognition, which means that representing and adapting to the future is not uniquely human (Osman, 2014).

### Conflict of interest statement

The author declares that the research was conducted in the absence of any commercial or financial relationships that could be construed as a potential conflict of interest.
